# Patterns of clozapine use, misuse and disuse in a mental health area in southern Spain

**DOI:** 10.1192/j.eurpsy.2022.1834

**Published:** 2022-09-01

**Authors:** L.I. Muñoz-Manchado, J.M. Mongil-Sanjuan, J.M. Pascual Paño, C. Rodríguez-Gómez, R. Torrecilla-Olavarrieta, J.I. Pérez-Revuelta, F. González-Saiz, J.M. Villagrán-Moreno

**Affiliations:** 1 UGC North of Cadiz, Mental Health Inpatient Unit, General Hospital, Jerez de la Frontera, Spain; 2 UGC North of Cadiz, Mental Health Inpatient Unit, General Hospital, Jerez De La Frontera, Spain

**Keywords:** clozapine, Patterns of use, PSYCHOTIC DISORDERS

## Abstract

**Introduction:**

Evidence supports clozapine as the best treatment in terms of efficacy, effectiveness and well-being, and as the gold standard in treatment-resistant psychotic disorders. Clozapine remains still underused, suffering initiation delays from 1.1 to 9.7 years. Furthermore, there is a scarcity of data about patterns of use, showing high variability worldwide (0.6-189.2/100. 000 inhabitants).

**Objectives:**

The main objective of this work is to carry out an analysis of the use of clozapine in our mental health catchment area. Thus, off-label use, the percentage of patients with clozapine depending on diagnosis, age and sex, and its use in mono and polytherapy are established. Besides, dosage and time between the first contact and the start of treatment with clozapine are recorded.

**Methods:**

A descriptive study has been developed on the patients with clozapine who consulted in the catchment area of the Jerez Mental Health Service between 2018 and 2019. Data were extracted from medical records.

**Results:**

From our population of 456.752 inhabitants, 449 patients received clozapine. 278 (61.9%) had a schizophrenia diagnosis; 33 (7.3%) delusional disorder and 34 (7,6%) schizoaffective disorder. The off-label use of clozapine was 19,1 %. The average mean dose used was 246,2 mg/day and 59% of the patients on clozapine were on polytherapy. Only 14,7% of these patients had a previous trial with clozapine on monotherapy.

**Conclusions:**

Rates of polytherapy, previous trials of clozapine monotherapy, off label use, rates of discontinuation and other variables are to be considered to precisely map the adequate use of clozapine in clinical settings.

**Disclosure:**

No significant relationships.

